# Sex hormone-binding globulin and arthritis: a Mendelian randomization study

**DOI:** 10.1186/s13075-020-02202-2

**Published:** 2020-05-18

**Authors:** Zihao Qu, Jiawei Huang, Fangkun Yang, Jianqiao Hong, Wei Wang, Shigui Yan

**Affiliations:** 1 Department of Orthopedic Surgery, The Second Affiliated Hospital, Zhejiang University School of Medicine, Zhejiang, 310009 Hangzhou China; 2 Orthopedic Research Institute of Zhejiang University, Zhejiang, 310009 Hangzhou China; 3 Spine Lab, Department of Orthopedic Surgery, The First Affiliated Hospital, Zhejiang University School of Medicine, Hangzhou, China; 4 Department of Cardiology, The Second Affiliated Hospital, Zhejiang University School of Medicine, Zhejiang, 310009 Hangzhou China

**Keywords:** Sex hormone-binding globulin, Osteoarthritis, Rheumatoid arthritis, Ankylosing spondylitis, Mendelian randomization

## Abstract

**Background:**

Sex hormone-binding globulin (SHBG) has been reported to be a risk factor associated with the development of arthritis by previous observational studies more so of three common forms of arthritis: osteoarthritis (OA), rheumatoid arthritis (RA), and ankylosing spondylitis (AS). This study aimed to determine whether the concentrations of circulating SHBG are causally associated with the risk of OA, RA, and AS.

**Methods:**

The two-sample Mendelian randomization (MR) approach was used for this study. The inverse-variance-weighted (IVW) method was used for the main analysis. Single-nucleotide polymorphisms (SNPs) associated with SHBG were selected from a large genome-wide association study (GWAS) of 28,837 European individuals. The summary statistics for OA, RA, and AS were extracted from the UK Biobank Resource (*n* = 361,141) and a GWAS dataset (*n* = 455,221).

**Results:**

Positive causal associations were found between circulating SHBG concentrations and OA (effect = 1.086; 95% CI, 1.009 to 1.168; *P* = 0.027) and RA (effect = 1.003; 95% CI, 1.000 to 1.007; *P* = 0.047) in overall analyses. However, there was no evidence of association between SHBG levels and AS. Based on the stratification of skeletal sites, SHBG levels were found to be significantly associated with hip OA (effect = 1.423; 95% CI, 1.219 to 1.660; *P* = 7.753 × 10^−6^). However, this was not the case with knee OA.

**Conclusions:**

There were positive causal effects of circulating SHBG on the development of OA and RA. Moreover, there was a site-specific association between SHBG and hip OA. Evidently, measurement of SHBG in serum could be valuable in the clinical assessment of arthritis especially in early screening and prevention of OA and RA. However, the mechanisms by which SHBG plays causal roles in the development of arthritis require further investigations.

## Background

Arthritis is a degenerative disease that affects people’s joints. Its symptoms include joint swelling, pain, activity limitation, and increase of skin temperature [1]. Based on the symptoms, blood tests, and imaging examinations, arthritis is clinically classified into multiple types [2]. Among them, osteoarthritis (OA) is the most common joint disorder which mainly affects the hand, knee, and hip [3]. As affected individuals grow older, OA results in the loss of function and chronic pain which continuously increase thus negatively impacting their quality of life [4]. Traditional treatment of OA includes conservative treatment and joint replacement [5, 6]. Despite the disease lacking a prevention approach, risk factors associated with its occurrence and progression are still being studied. Other types of arthritis that are usually triggered by autoimmune inflammation such as rheumatoid arthritis (RA) and ankylosing spondylitis (AS) are similar to OA in regards to their progression. They also lead to chronic pain and degeneration of joints [7]. RA have been treated as an autoimmune disease because of its persistent synovitis and systemic inflammation [7]. Women and the elderly are the susceptible population to RA. RA occurs thrice more frequently in women than in men [8, 9]. AS is also characterized by systematic inflammation. It mostly affects the axial skeleton in young men manifesting as inflammatory back pain, enthesitis, and asymmetrical peripheral oligoarthritis. The pathogenesis and effective treatment of AS are currently controversial [10]. Researchers largely attribute the progression of RA and AS to genetic factors.

Previous studies have reported various risk factors associated with arthritis to help further understand the cause of arthritis. For instance, females [11], increase of age [12], joint surgery [13], and obesity [14] are related to the higher incidence of OA. In the same line, genetic factors [15], smoking [16], low consumption of alcohol [17], and high coffee intake [18] are related to the higher incidence of RA. Although AS is suggested to be a highly heritable joint disease [19], some environmental risk factors are proposed to be involved in its development [20]. Besides these factors, the sex hormone-binding globulin (SHBG) has been reported to be a risk factor of these three types of arthritis [21, 22]. SHBG is a serum protein that has high affinity to bind with circulating sex hormones. Variation of SHBG concentration causes a series of physiological and pathological effects in the human body [23, 24]. Multiple randomized controlled trials (RCTs) suggest that SHBG level is associated with OA, RA, and AS [21, 22]. Nevertheless, there is still inconformity among the results of these studies. The potential interactions between SHBG concentrations and arthritis remain unclear. As such, a novel research methodology is needed.

Mendelian randomization (MR) approach has been widely used in recent years to determine the potential causal effects of exposures on clinical outcomes. According to Mendel’s second law, genetic variants are randomly allocated at conception. MR studies use single-nucleotide polymorphisms (SNPs) as instrumental variables to avoid the reverse causation and confounding factors which cause bias in traditional observational studies [25, 26]. MR method has been applied to estimate the causal associations between body mass index (BMI) [27], bone mineral density (BMD) [28], smoking [29] and coffee intake [30], and OA. In addition, robust causal associations of interleukin-6 [31], blood magnesium [32], and vitamin D [33] with RA have been demonstrated by previous MR approach-based studies. However, the role of circulating SHBG in arthritis has not been well established by these studies.

Herein, the MR approach was used to analyze the potential causal effect of circulating SHBG concentrations on the risk of contracting OA, RA, and AS. The site-specific associations between SHBG level and OA as well as the sex-specific associations between SHBG level and three types of arthritis were further explored.

## Methods

### Selection of genetic variants

Instrumental variables associated with circulating SHBG concentration were extracted from a large GWAS dataset [34], which included 28,837 individuals (13,899 women and 14,938 men) of European ancestry. SNPs associated with SHBG level at genome-wide significance (*P* < 5 × 10^−8^) were utilized. The corresponding linkage disequilibrium (Supplementary Table 1) was tested on the LD-link website (https://ldlink.nci.nih.gov/, European; *r*^2^ < 0.1). Adjustments were made for sex and BMI. Thirteen SNPs were finally identified as genetic variants. The characteristics of the selected SNPs for SHBG concentration are displayed in Table 1.

**Table 1:** Characteristics of SNPs for circulating SHBG concentration from GWAS meta-analysis

Gene	SNP	Chromosome: position (hg19)	EA	Association with exposure	
				*β* (SE)	*P* value
*PRMT6*	rs17496332	1: 107546375	G	0.028 (0.0041)	1.40E−11
*GCKR*	rs780093	2: 27742603	C	0.032 (0.0039)	2.20E−16
*BAIAP2L1*	rs3779195	7: 97993362	T	0.028 (0.0051)	2.70E−08
*ZBTB10*	rs440837	8: 81461974	G	0.028 (0.0047)	3.40E−09
*JMJD1C*	rs7910927	10: 65138910	G	0.048 (0.0039)	6.10E−35
*SLCO1B1*	rs4149056	12: 21331549	T	0.029 (0.0052)	1.90E−08
*NR2F2*	rs8023580	15: 96708291	C	0.030 (0.0044)	8.30E−12
*ZNF652*	rs2411984	17: 47445751	A	0.033 (0.0044)	3.50E−14
*SHBG*	rs12150660	17: 7521915	T	0.103 (0.0047)	1.80E−106
*SHBG*	rs6258	17: 7534678	T	− 0.272 (0.017)	1.03E−60
*SHBG*	rs1641537	17: 7545721	C	0.081 (0.0062)	8.19E−39
*TP53*	rs1625895	17: 7578115	C	0.052 (0.0067)	1.17E−14
*TDGF3*	rs1573036	X: 109820068	T	0.028 (0.0037)	4.10E−14

*Gene* nearest gene to SHBG associated SNP, *EA* effect allele, *β* per allele effect on SHBG levels, *SE* standard error, *P value P* value for the genetic association

### Genetic associations with arthritis

Summary statistics for the effect of SHBG-associated SNPs on OA were derived from a large GWAS meta-analysis that included 455,221 European individuals [35]. Based on joint morphology, hip and knee are the established risk factors for OA [36, 37]. In cognizant of this, the summary data for hip and knee OA were also extracted from the GWAS dataset above, which contained 393,873 and 403,124 European individuals, respectively. The corresponding summary statistics for OA, RA, and AS were obtained from the UK Biobank Resource to investigate the impact of sex difference on causal associations between SHBG and arthritis. 361,141 participants of European ancestry were identified. Among them, 194,153 were women while 166,988 were men. There were 30,046 cases of OA (19,397 women and 10,649 men), 4017 cases of RA (2758 women and 1259 men), and 1038 cases of AS (367 women and 671 men). The characteristics of GWAS studies of the included data are showed in Supplementary Table 2. The summary data for the overall analyses of RA and AS were also extracted from the UK Biobank. All studies contributing data for analyses were approved by relevant ethics committees. Moreover, all participants provided written informed consents prior to the study.

### Statistical analysis

The MR approach was used to identify the potential causal link of SHBG concentrations with OA, RA, and AS. Estimates for the genetically causal associations were obtained by applying the fixed-effect inverse-variance-weighted (IVW) analysis. This was done to establish the overall effect of each concentration of SHBG on the outcomes. For MR analyses, a statistical significance threshold of *P* < 0.05 was used. The statistical power was calculated based on the mRnd website (https://shiny.cnsgenomics.com/mRnd/). Type-I error rate was set to 0.05. The selected instrumental variables explained 8% of the variance in circulating SHBG levels. In the analyses of SHBG levels, our study had 100% power to detect an odds ratio (OR) of 1.1 for overall OA and an OR of 1.5 for hip OA. And we had 47% power to detect an OR of 1.1 for knee OA. However, the power to detect an OR for the other outcomes in the analyses of SHBG levels was lower than 10%. This might due to the low proportion of cases in the datasets from UK Biobank.

A weighted median method was used to examine the median value of the ratio instrumental variable estimates in the sensitivity analysis. This was done as a complement to IVW analysis. The MR-Egger method was performed to account for potential pleiotropy of the genetic variants which would have influenced the outcome through pathways other than the exposure as this could have caused bias to the analytical results [38]. The MR pleiotropy residual sum and outlier (MR-PRESSO) method were also used to test for horizontal pleiotropy. The outlying genetic variants were identified by applying this method. In addition, traits associated with SHBG-associated SNPs were searched on the PhenoScanner V2 website to determine whether the selected instrumental variables were related to any confounder in the potential associations between SHBG levels and the three types of arthritis. As such, in the sensitivity analysis, IVW analysis was performed after removing the genetic variant rs780093, which was found to be related with some confounders after searching in the PhenoScanner website. The *P* value of the association between rs12150660 and hip OA was lower than 1.00 × 10^−4^ (*P* = 1.45 × 10^−5^). In cognizant of this, IVW analysis was performed on hip OA after removing this SNP (rs12150660) from instrumental variables. This was done to test the robustness of the causal association between SHBG levels and hip OA.

All statistical analyses were conducted using R version 3.6.1 and the R package “MendelianRandomization.”

## Results

The associations between genetic variants for circulating SHBG concentrations and all the outcomes are summarized in Supplementary Table 3–6. The potential causal effects of SHBG concentrations on the three types of arthritis (OA, RA, and AS) were estimated by IVW analyses. Based on a search done in the PhenoScanner website, none of the selected instrumental variables was strongly associated with traditional risk factors of OA, RA, and AS except rs780093. It was found to be related to body weight. However, rs17496332, rs7910927, rs12150660, rs1641537, and rs1573036 were found to be correlated with traits which have been reported recently to be related to OA, RA, or AS. The other remaining genetic variants were not associated with any confounders (Supplementary Table 7). For instance, rs780093 and rs7910927 are associated with triglyceride levels while rs12150660 and rs1573036 are associated with testosterone levels.

### Causal association with overall OA, RA, and AS

**Fig. 1:**
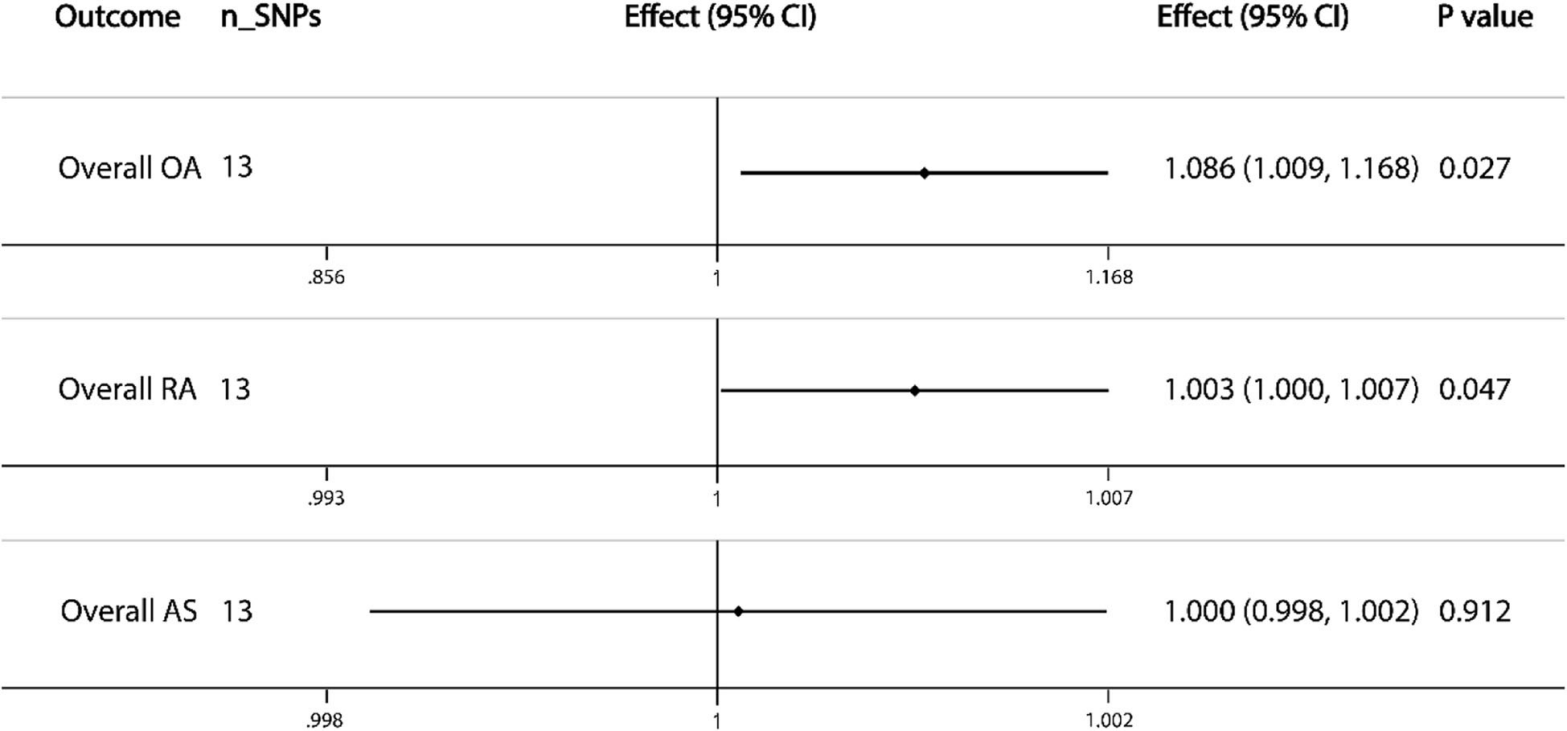
Causal associations between SHBG concentrations and overall OA, RA, and AS. The number of genetic variants, effects, 95% confidence intervals, and *P* values of associations are contained. n_SNPs, the number of SNPs used as instrumental variables; Effect, the combined causal effect; CI, confidence interval; *P* value, *P* value of the causal estimate

The overall causal association between SHBG levels and OA (effect = 1.086; 95% CI, 1.009 to 1.168; *P* = 0.027) and RA (effect = 1.003; 95% CI, 1.000 to 1.007; *P* = 0.047) was significant. However, no association was found between SHBG levels and AS (Fig. 1). These results indicated that SHBG concentrations were positively associated with the risk of OA and RA in all individuals.

### Effect of sex on SHBG association with OA, RA, and AS

**Fig. 2:**
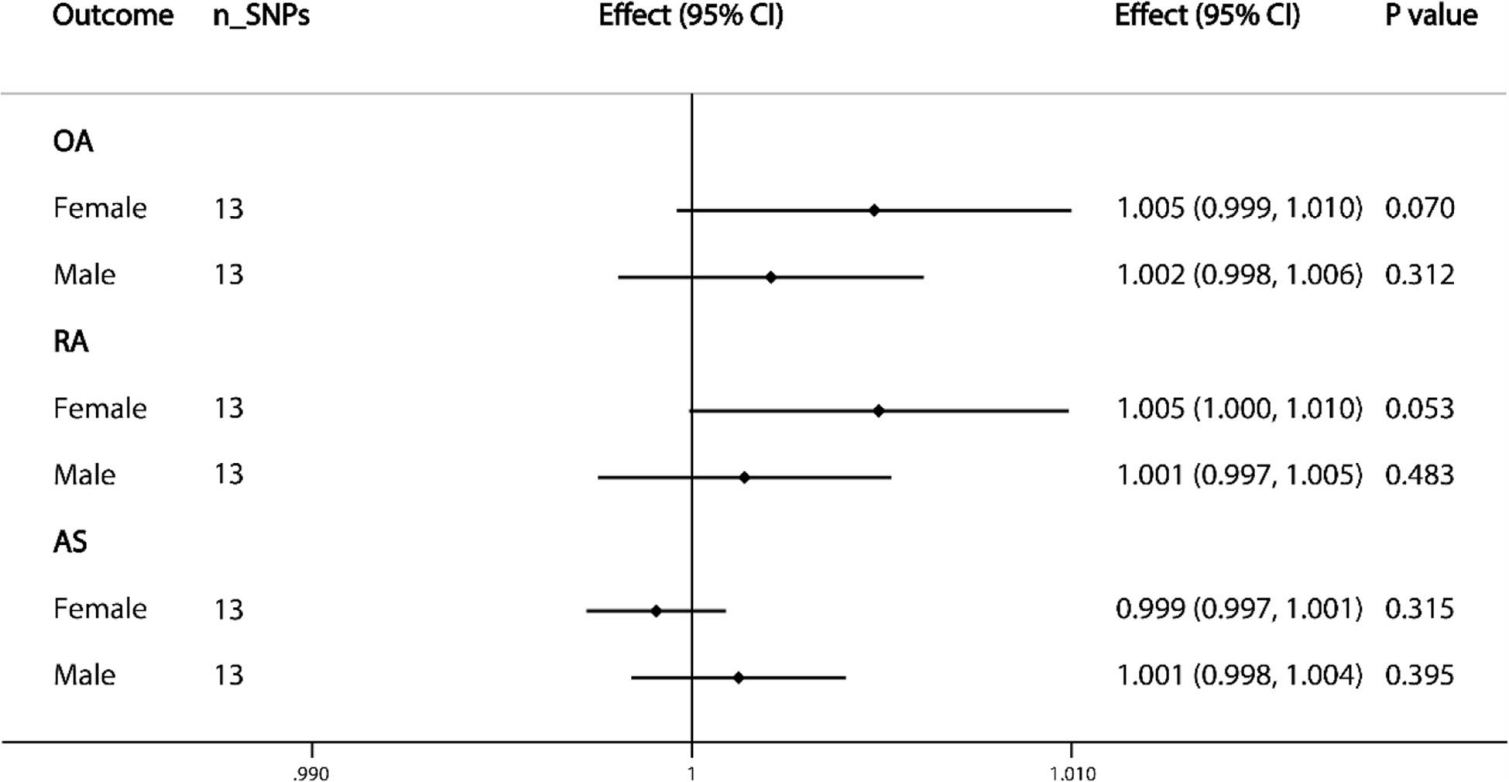
Causal associations between SHBG concentrations and OA, RA, and AS by sex. The number of genetic variants, effects, 95% confidence intervals, and *P* values of associations are contained. Two estimates under each subtitle represent the causal effect of SHBG levels on the corresponding type of arthritis in women and men, respectively. n_SNPs, the number of SNPs used as instrumental variables; Effect, the combined causal effect; CI, confidence interval; *P* value, *P* value of the causal estimate

Little evidence was provided for the sex-specific causal effect of SHBG levels on the three types of arthritis. However, there was a significant effect of sex on the association between SHBG levels and the risk of OA (*P* = 3.68 × 10^−3^ for sex difference) and RA (*P* = 2.95 × 10^−4^ for sex difference) based on the Student *t* test. The estimates of combined causal effects of genetically predicted SHBG concentration on OA, RA, and AS of different sexes are integrated in Fig. 2.

### Causal association with OA by site

There was a significant causal association between levels of SHBG and hip OA (effect = 1.423; 95% CI, 1.219 to 1.660; *P* = 7.753 × 10^−6^). However, there was little evidence of association between SHBG levels and knee OA (Fig. 2).

### Sensitivity analysis

**Fig. 3:**
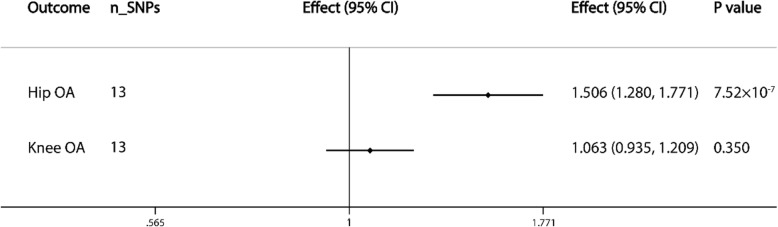
Causal associations between SHBG concentrations and hip and knee OA. The number of genetic variants, effects, 95% confidence intervals, and *P* values of associations are contained. n_SNPs, the number of SNPs used as instrumental variables; Effect, the combined causal effect; CI, confidence interval; *P* value, *P* value of the causal estimate

The estimates of sensitivity analysis based on IVW analyses were consistent with those obtained after applying the weighted median, MR-Egger, and MR-Egger intercept methods. However, in the weighted median analysis for the causal effect of circulating SHBG levels on all OA patients (*P* = 0.247) and female OA patients (*P* = 0.014), the significance was in the opposite direction compared with that of IVW analysis. All the intercept values of MR-Egger did not differ from zero. These results strongly suggested that the observed associations were not biased by pleiotropic effects (Supplementary Table 8). rs17496332 was the only outlying SNP in the association between serum SHBG levels and knee OA based on MR-PRESSO analysis (Supplementary Table 9). After removing this genetic variant (rs17496332), the results were similar with those of the analysis including all SNPs (effect = 1.009; 95% CI, 0.854 to 1.192; *P* = 0.917). In addition, when rs780093 was excluded, the *P* value of the association between SHBG levels and all OA patients became bigger (*P* = 0.050). There was also a significant causal association between SHBG levels and OA (effect = 1.006; 95% CI, 1.000 to 1.011; *P* = 0.032) and RA (effect = 1.006; 95% CI, 1.001 to 1.011; *P* = 0.025) in female patients (Supplementary Table 10). Moreover, a similar association between SHBG levels and hip OA (effect = 1.256; 95% CI, 1.035 to 1.526; *P* = 0.021) was observed when rs12150660 (was found to be associated with hip OA) was removed (Fig. 3).

## Discussion

This MR study suggested positive causal associations between circulating SHBG concentrations and the risk of OA and RA in all individuals. However, there were no causal associations between circulating SHBG concentrations and the risk of AS. Stratified analyses suggested that the potential effects of SHBG levels on OA and RA in women were more significant than that in men. In addition, a positive association between SHBG levels and hip OA was observed.

In line with these findings, previous studies also suggest that higher SHBG concentration was a risk factor of OA. Lower concentrations of SHBG were found to reduce the risk of hip OA as well as total hip replacement [39]. In addition, SHBG was reported to be positively associated with tibial cartilage volume [40]. A randomized controlled trial demonstrated a statistically positive association between SHBG levels and patella bone volume [41]. However, lower SHBG concentrations were also detected in middle-aged women diagnosed with OA [22], which was a contrary report from that of our study. Significantly lower levels of SHBG were detected in RA patients compared to their controls in an observational study [42]. However, a recent study by Tengstrand et al. suggested that there were no differences in SHBG levels of RA patients and their controls [43]. In a clinical trial by Navarro et al., lower concentrations of SHBG in serum were observed in a group of RA patients undergoing glucocorticoid treatment compared to the group without treatment [44]. This was an indication that SHBG level was positively associated with the severity of RA. These results were consistent with those of this study. No association of SHBG with AS was found in our findings. These results were inconsistent with those reported by other studies. For instance, a study in the UK by Mitra et al. reported that there were lower concentrations of serum SHBG in AS patients compared to their controls [45]. In the same line, Giltay et al. reported that serum SHBG levels in AS patients and their controls were not significantly different [46].

This study suggested a potential sex difference in causal associations between SHBG concentrations and OA and RA. The sex difference in the prevalence of OA and RA has been reported by multiple studies and clinical guidelines [47, 48]. Women were more than men among the patients diagnosed with OA. Further to this, being female has also been included to be a risk factor associated with RA.

In this study, we investigated the potential association between genetically predicted SHBG concentrations and three types of arthritis (OA, RA, and AS). The two-sample MR analysis was applied using SNPs from corresponding summary statistics of GWAS datasets as instrumental variables. This MR approach allowed for the estimation of the causal effect of SHBG levels on outcomes with a large sample size and at high precision. This prevented drawbacks of the conventional observational studies such as reverse causality and confounding factors after conception. Moreover, the pleiotropic effects in this study were detected by applying the MR-Egger method. This minimized the effects of single SNPs on the phenotypes or downstream pathways that could affect the outcome by causing confounded estimates. In addition, only European participants were included in this study to minimize bias of the genetic variants’ frequencies that could arise if different population subgroups were included.

Nevertheless, this study was limited by several factors. There were inconsistencies between the results of IVW and weighted median analyses which could have been caused by the potential differences in validities of all the SNPs. In addition, the estimated effect of the positive association between SHBG concentrations and the risk of RA was weak thus influencing the robustness of the analytical results. Considering the Bonferroni correction of multiple independent tests, the statistical significance should be defined as a *P* value of < 0.005 (one exposure and 11 outcomes). Therefore, our findings except the association between SHBG levels and hip OA were deemed suggestive evidence of possible associations (0.005 < *P* < 0.05). Another limiting factor was the fact that only European individuals were included in this study. This necessitates further studies incorporating populations from different races for more conclusive results.

## Conclusions

This MR study indicates that circulating SHBG concentrations are positively associated with the risk of OA and RA. And SHBG levels are positively associated with hip OA. Nevertheless, there is little evidence for the causal effect of SHBG on AS. Significant sex differences in associations between SHBG and the risk of OA and RA were observed. Evidently, this study highlights the potential role of SHBG in the development of RA and OA of specific skeletal sites. Cognizant to this, measurement of SHBG in serum could be valuable in the clinical assessment of arthritis especially in early screening and prevention of OA and RA.

## Supplementary information

**Additional file 1: Table S1.** Calculation of linkage disequilibrium of selected SNPs. **Table S2.** The characteristics of GWAS studies on the included outcomes. **Table S3.** The association information of SHBG SNPs with OA in all participants and single sex. **Table S4.** The association information of SHBG SNPs with RA in all participants and single sex. **TableS5.** The association information of SHBG SNPs with AS in all participants and single sex. **Table S6.** The association information of SHBG SNPs with OA in two skeletal sites. **Table S7.** Related traits of SHBG SNPs. **Table S8.** Weighted median and MR-Egger analysis for genetic associations between serum SHBG concentration and all the outcomes. **Table S9.** MR-PRESSO analysis for genetic associations between serum SHBG concentration and all the outcomes

## Data Availability

The summary statistics of the UK Biobank and GWAS datasets used in this study are available on request provided there is a clear statement of purpose.

## Abbreviations

SHBG: Sex hormone-binding globulin
OA: Osteoarthritis
RA: Rheumatoid arthritis
AS: Ankylosing spondylitis
MR: Mendelian randomization
SNP: Single-nucleotide polymorphism
GWAS: Genome-wide association study
RCT: Randomized controlled trail
BMI: Body mass index
BMD: Bone mineral density
IVW: Inverse-variance-weighted
OR: Odds ratio
MR-PRESSO: Mendelian randomization pleiotropy residual sum and outlier
