# Supplementary material for: Sex hormone-binding globulin and arthritis: a Mendelian randomization study
Source: Arthritis Res Ther. 2020 May 18;22:118. doi: 10.1186/s13075-020-02202-2 (PMC7236473; doi:10.1186/s13075-020-02202-2)
Supplement: Supplementary file 1 — Additional file 1: Table S1. Calculation of linkage disequilibrium of selected SNPs. Table S2. The characteristics of GWAS studies on the included outcomes. Table S3. The association information of SHBG SNPs with OA in all participants and single sex. Table S4. The association information of SHBG SNPs with RA in all participants and single sex. TableS5. The association information of SHBG SNPs with AS in all participants and single sex. Table S6. The association information of SHBG SNPs with OA in two skeletal sites. Table S7. Related traits of SHBG SNPs. Table S8. Weighted median and MR-Egger analysis for genetic associations between serum SHBG concentration and all the outcomes. Table S9. MR-PRESSO analysis for genetic associations between serum SHBG concentration and all the outcomes [file 13075_2020_2202_MOESM1_ESM.docx]

Supplementary Material

Table 1. Calculation of linkage disequilibrium of selected SNPs

| RS number | rs12150660 | rs6258 | rs1641537 | rs1625895 | rs2411984 |
| --- | --- | --- | --- | --- | --- |
| rs12150660 | 1.0 | 0.003 | 0.035 | 0.004 | 0.003 |
| rs6258 | 0.003 | 1.0 | 0.001 | 0.001 | 0.003 |
| rs1641537 | 0.035 | 0.001 | 1.0 | 0.0 | 0.0 |
| rs1625895 | 0.004 | 0.001 | 0.0 | 1.0 | 0.002 |
| rs2411984 | 0.003 | 0.003 | 0.0 | 0.002 | 1.0 |

The 5 SNPs in the table are on the same chromosome.

Population = (CEU) Utah Residents from North and West Europe; r^2^ < 0.1.

Table 2. The characteristics of GWAS studies on the included outcomes

| Outcome | SNPs | Consortium | Total population | Cases/controls | Ethnicity | References |
| --- | --- | --- | --- | --- | --- | --- |
| Overall OA | 13 | UK Biobank and arcOGEN | 455221 | 77052/378169 | European | Identification of New Therapeutic Targets for Osteoarthritis Through Genome-Wide Analyses of UK Biobank Data |
| Hip OA | 13 | UK Biobank and arcOGEN | 393873 | 15704/378169 | European | Identification of New Therapeutic Targets for Osteoarthritis Through Genome-Wide Analyses of UK Biobank Data |
| Knee OA | 13 | UK Biobank and arcOGEN | 403124 | 24955/378169 | European | Identification of New Therapeutic Targets for Osteoarthritis Through Genome-Wide Analyses of UK Biobank Data |
| OA in female | 13 | UK Biobank | 194153 | 19397/174756 | European | UK Biobank (Neale lab) |
| OA in male | 13 | UK Biobank | 166988 | 10649/156339 | European | UK Biobank (Neale lab) |
| Overall RA | 13 | UK Biobank | 361141 | 4017/357124 | European | UK Biobank (Neale lab) |
| RA in female | 13 | UK Biobank | 194153 | 2758/191395 | European | UK Biobank (Neale lab) |
| RA in male | 13 | UK Biobank | 166988 | 1259/165729 | European | UK Biobank (Neale lab) |
| Overall AS | 13 | UK Biobank | 361141 | 1038/360103 | European | UK Biobank (Neale lab) |
| AS in female | 13 | UK Biobank | 194153 | 367/193786 | European | UK Biobank (Neale lab) |
| AS in male | 13 | UK Biobank | 166988 | 671/166317 | European | UK Biobank (Neale lab) |

SNPs represents the number of SNPs selected as instrumental variables for serum SHBG concentration in analysis with each outcome.

Table 3. The association information of SHBG SNPs with OA in all participants and single sex

| SNP | EA | Overall OA | | OA in female | | OA in male | |
| --- | --- | --- | --- | --- | --- | --- | --- |
|  |  | β(SE) | P value | β(SE) | P value | β(SE) | P value |
| rs17496332 | G | 0.019 (0.0058) | 0.0011 | 9.21E-05 (4.02E-04) | 0.0007 | 1.78E-04 (3.17E-04) | 0.8134 |
| rs780093 | C | 0.0081 (0.0057) | 0.1531 | -4.92E-04 (3.91E-04) | 0.1759 | 2.36E-06 (3.08E-04) | 0.3401 |
| rs3779195 | T | -0.013 (0.0071) | 0.0664 | 8.51E-04 (4.89E-04) | 0.1648 | 1.06E-04 (3.84E-04) | 0.8133 |
| rs440837 | G | -0.0044 (0.0068) | 0.5206 | 3.09E-04 (4.71E-04) | 0.4482 | 4.22E-04 (3.73E-04) | 0.3111 |
| rs7910927 | G | 0.0098 (0.0055) | 0.0757 | 1.09E-04 (3.80E-04) | 0.0538 | 5.27E-04 (3.00E-04) | 0.7501 |
| rs4149056 | T | -0.0016 (0.0077) | 0.8319 | -4.83E-04 (5.31E-04) | 0.9348 | -1.89E-04 (4.17E-04) | 0.0047 |
| rs8023580 | C | -0.001 (0.0062) | 0.8689 | 2.70E-04 (4.26E-04) | 0.1474 | -6.99E-04 (3.36E-04) | 0.7171 |
| rs2411984 | A | -0.0076 (0.0062) | 0.2166 | -9.26E-05 (4.12E-04) | 0.6384 | 3.97E-04 (3.25E-04) | 0.4782 |
| rs12150660 | T | 0.021 (0.0064) | 0.0010 | 0.0011 (4.38E-04) | 0.2947 | -6.57E-05 (3.45E-04) | 0.8716 |
| rs6258 | T | 0.0059 (0.0335) | 0.8600 | -0.0022 (0.0058) | 0.7009 | -0.0069 (0.0051) | 0.1772 |
| rs1641537 | C | -0.0095 (0.0085) | 0.2608 | -2.05E-04 (5.83E-04) | 0.4798 | 1.26E-04 (4.59E-04) | 0.0664 |
| rs1625895 | C | -0.0023 (0.0083) | 0.7866 | -1.70E-04 (5.75E-04) | 0.7751 | -3.87E-04 (4.53E-04) | 0.5847 |
| rs1573036 | T | -0.0009 (0.0048) | 0.8459 | 5.18E-04 (3.89E-04) | 0.2826 | 2.97E-04 (2.17E-04) | 0.1633 |

EA = effect allele; β = per allele effect on SD units; SE = standard error; P value = p-value for the genetic association.

Table 4. The association information of SHBG SNPs with RA in all participants and single sex

| SNP | EA | Overall RA | | RA in female | | RA in male | |
| --- | --- | --- | --- | --- | --- | --- | --- |
|  |  | β(SE) | P value | β(SE) | P value | β(SE) | P value |
| rs17496332 | G | 1.33E-04 (2.61E-04) | 0.6101 | 9.21E-05 (4.02E-04) | 0.8188 | 1.78E-04 (3.17E-04) | 0.5739 |
| rs780093 | C | -2.62E-04 (2.54E-04) | 0.3009 | -4.92E-04 (3.91E-04) | 0.2086 | 2.36E-06 (3.08E-04) | 0.9939 |
| rs3779195 | T | 5.06E-04 (3.17E-04) | 0.1101 | 8.51E-04 (4.89E-04) | 0.0818 | 1.06E-04 (3.84E-04) | 0.7831 |
| rs440837 | G | 3.59E-04 (3.07E-04) | 0.2414 | 3.09E-04 (4.71E-04) | 0.5117 | 4.22E-04 (3.73E-04) | 0.2573 |
| rs7910927 | G | 3.02E-04 (2.47E-04) | 0.2221 | 1.09E-04 (3.80E-04) | 0.7743 | 5.27E-04 (3.00E-04) | 0.0791 |
| rs4149056 | T | -3.47E-04 (3.44E-04) | 0.3142 | -4.83E-04 (5.31E-04) | 0.3635 | -1.89E-04 (4.17E-04) | 0.6505 |
| rs8023580 | C | -1.75E-04 (2.77E-04) | 0.5266 | 2.70E-04 (4.26E-04) | 0.5252 | -6.99E-04 (3.36E-04) | 0.0377 |
| rs2411984 | A | 1.35E-04 (2.68E-04) | 0.6144 | -9.26E-05 (4.12E-04) | 0.8222 | 3.97E-04 (3.25E-04) | 0.2224 |
| rs12150660 | T | 5.43E-04 (2.84E-04) | 0.0561 | 0.0011 (4.38E-04) | 0.0149 | -6.57E-05 (3.45E-04) | 0.8490 |
| rs6258 | T | -6.36E-04 (0.0015) | 0.6725 | -0.0018 (0.0023) | 0.4333 | 7.75E-04 (0.0018) | 0.6708 |
| rs1641537 | C | -5.27E-05 (3.78E-04) | 0.8893 | -2.05E-04 (5.83E-04) | 0.7246 | 1.26E-04 (4.59E-04) | 0.7831 |
| rs1625895 | C | -2.74E-04 (3.73E-04) | 0.4633 | -1.70E-04 (5.75E-04) | 0.7675 | -3.87E-04 (4.53E-04) | 0.3934 |
| rs1573036 | T | 3.78E-04 (2.09E-04) | 0.0701 | 5.18E-04 (3.89E-04) | 0.1827 | 2.97E-04 (2.17E-04) | 0.1703 |

EA = effect allele; β = per allele effect on SD units; SE = standard error; P value = p-value for the genetic association.

Table 5. The association information of SHBG SNPs with AS in all participants and single sex

| SNP | EA | Overall AS | | AS in female | | AS in male | |
| --- | --- | --- | --- | --- | --- | --- | --- |
|  |  | β(SE) | P value | β(SE) | P value | β(SE) | P value |
| rs17496332 | G | -1.42E-04 (1.33E-04) | 0.2884 | -2.92E-04 (1.48E-04) | 0.0482 | 3.08E-05 (2.32E-04) | 0.8944 |
| rs780093 | C | -3.44E-04 (1.30E-04) | 0.0080 | -1.94E-04 (1.44E-04) | 0.1763 | -5.18E-04 (2.25E-04) | 0.0214 |
| rs3779195 | T | 6.86E-05 (1.62E-04) | 0.6721 | -1.18E-04 (1.80E-04) | 0.5125 | 2.88E-04 (2.81E-04) | 0.3042 |
| rs440837 | G | -1.38E-04 (1.57E-04) | 0.3771 | -1.27E-04 (1.73E-04) | 0.4640 | -1.52E-04 (2.73E-04) | 0.5769 |
| rs7910927 | G | -8.46E-05 (1.26E-04) | 0.5030 | -1.13E-05 (1.40E-04) | 0.9358 | -1.72E-04 (2.20E-04) | 0.4344 |
| rs4149056 | T | 2.00E-04 (1.76E-04) | 0.2567 | 2.23E-04 (1.95E-04) | 0.2528 | 1.74E-04 (3.05E-04) | 0.5686 |
| rs8023580 | C | -8.50E-05 (1.41E-04) | 0.5479 | -1.10E-04 (1.56E-04) | 0.4817 | -5.39E-05 (2.46E-04) | 0.8267 |
| rs2411984 | A | -4.73E-05 (1.37E-04) | 0.7297 | -1.11E-04 (1.51E-04) | 0.4621 | 2.80E-05 (2.38E-04) | 0.9065 |
| rs12150660 | T | 2.54E-04 (1.45E-04) | 0.0809 | 3.28E-05 (1.61E-04) | 0.8383 | 5.05E-04 (2.53E-04) | 0.0455 |
| rs6258 | T | 6.45E-04 (7.69E-04) | 0.4016 | 1.37E-05 (8.53E-04) | 0.9872 | 0.0014 (0.0013) | 0.3072 |
| rs1641537 | C | 1.18E-05 (1.93E-04) | 0.9513 | -3.36E-04 (2.14E-04) | 0.1165 | 4.11E-04 (3.36E-04) | 0.2217 |
| rs1625895 | C | 2.25E-04 (1.91E-04) | 0.2391 | 3.24E-04 (2.11E-04) | 0.1245 | 1.03E-04 (3.31E-04) | 0.7548 |
| rs1573036 | T | 3.44E-05 (1.07E-04) | 0.7472 | 5.94E-05 (1.43E-04) | 0.6773 | 2.10E-05 (1.59E-04) | 0.8946 |

EA = effect allele; β = per allele effect on SD units; SE = standard error; P value = p-value for the genetic association.

Table 6. The association information of SHBG SNPs with OA in two skeletal sites

| SNP | EA | Hip OA | | Knee OA | |
| --- | --- | --- | --- | --- | --- |
|  |  | β(SE) | P value | β(SE) | P value |
| rs17496332 | G | 0.0239 (0.0123) | 0.0521 | 0.0296 (0.0098) | 0.0026 |
| rs780093 | C | 0.0309 (0.0120) | 0.0100 | 0.0234 (0.0095) | 0.0143 |
| rs3779195 | T | -0.0022 (0.0150) | 0.8828 | -0.0239 (0.0119) | 0.0448 |
| rs440837 | G | -0.0164 (0.0145) | 0.2577 | 0.0012 (0.0115) | 0.9152 |
| rs7910927 | G | 0.0303 (0.0117) | 0.0095 | 0.0033 (0.0093) | 0.7202 |
| rs4149056 | T | -0.0073 (0.0162) | 0.6520 | 0.0061 (0.0129) | 0.6349 |
| rs8023580 | C | -0.0028 (0.0130) | 0.8302 | 0.0069 (0.0104) | 0.5054 |
| rs2411984 | A | -0.0095 (0.0137) | 0.4874 | -0.0085 (0.0106) | 0.4194 |
| rs12150660 | T | 0.0583 (0.0134) | 1.45E-05 | 0.0157 (0.0107) | 0.1437 |
| rs6258 | T | 0.0562 (0.0706) | 0.4256 | 0.0393 (0.0564) | 0.4853 |
| rs1641537 | C | 0.0220 (0.0179) | 0.2184 | -0.0322 (0.0142) | 0.0231 |
| rs1625895 | C | 0.0226 (0.0177) | 0.1999 | -0.0060 (0.0140) | 0.6676 |
| rs1573036 | T | 0.0021 (0.0106) | 0.8413 | -0.0002 (0.0082) | 0.9850 |

EA = effect allele; β = per allele effect on SD units; SE = standard error; P value = p-value for the genetic association.

Table 7. Related traits of SHBG SNPs.

| SNP | Chromosome: Position | Trait | Beta | P value |
| --- | --- | --- | --- | --- |
| rs17496332 | 1: 107546375 | Whole body fat mass | -0.014 | 1.27E-08 |
| rs17496332 | 1: 107546375 | Waist circumference | -0.013 | 2.93E-08 |
| rs780093 | 2: 27742603 | Triglycerides | 0.111 | 6.17E-220 |
| rs780093 | 2: 27742603 | Total cholesterol levels | 0.052 | 3.00E-47 |
| rs780093 | 2: 27742603 | Alcohol intake frequency | 0.048 | 1.21E-39 |
| rs780093 | 2: 27742603 | Fasting glucose | -0.028 | 2.40E-38 |
| rs780093 | 2: 27742603 | Weight | -0.017 | 1.39E-14 |
| rs780093 | 2: 27742603 | Type II diabetes | -0.080 | 1.30E-09 |
| rs780093 | 2: 27742603 | Low density lipoprotein | 0.022 | 2.36E-08 |
| rs3779195 | 7: 97993362 | NA | NA | NA |
| rs440837 | 8: 81461974 | NA | NA | NA |
| rs7910927 | 10: 65138910 | Triglycerides | -0.029 | 3.48E-11 |
| rs4149056 | 12: 21331549 | NA | NA | NA |
| rs8023580 | 15: 96708291 | NA | NA | NA |
| rs2411984 | 17: 47445751 | NA | NA | NA |
| rs12150660 | 17: 7521915 | Testosterone levels | -31.800 | 1.00E-41 |
| rs6258 | 17: 7534678 | NA | NA | NA |
| rs1641537 | 17: 7545721 | Weight | 0.018 | 4.25E-08 |
| rs1625895 | 17: 7578115 | NA | NA | NA |
| rs1573036 | X: 109820068 | Testosterone levels | 82.300 | 2.00E-22 |

The included traits were found to be related with SHBG-associated SNPs and were thought to be associated with the risk of OA, RA or AS. The results were obtained from PhenoScanner V2 website (http://www.phenoscanner.medschl.cam.ac.uk/).

Table 8. Weighted median and MR-Egger analysis for genetic associations between serum SHBG concentration and all the outcomes

| Method | Weighted median | MR-Egger | |
| --- | --- | --- | --- |
|  |  | Estimate | Intercept |
| Overall OA |  |  |  |
| Estimate (95% CI) | 0.066 (-0.046, 0.178) | 0.124 (-0.100, 0.347) | -0.002 (-0.013, 0.009) |
| P value | 0.247 | 0.278 | 0.670 |
| OA in female |  |  |  |
| Estimate (95% CI) | 0.009 (0.002, 0.016) | 0.008 (-0.003, 0.018) | -1.5E-04 (-6.7E-04, 3.7E-04) |
| P value | 0.014 | 0.169 | 0.571 |
| OA in male |  |  |  |
| Estimate (95% CI) | 6.96E-06 (-0.006, 0.006) | -4.4E-04 (-0.010, 0.009) | 1.3E-04 (-3.0E-04, 5.7E-04) |
| P value | 0.998 | 0.928 | 0.548 |
| Overall RA |  |  |  |
| Estimate (95% CI) | 0.005 (0.001, 0.009) | 0.003 (-0.004, 0.010) | 1.8E-05 (-3.0E-04, 3.4E-04) |
| P value | 0.026 | 0.372 | 0.911 |
| RA in female |  |  |  |
| Estimate (95% CI) | 0.008 (0.001, 0.015) | 0.007 (-0.002, 0.017) | -1.4E-04 (-6.2E-04, 3.4E-04) |
| P value | 0.029 | 0.132 | 0.559 |
| RA in male |  |  |  |
| Estimate (95% CI) | -1.7E-04 (-0.006, 0.005) | -0.002 (-0.010, 0.006) | 2.0E-04 (-1.8E-04, 5.7E-04) |
| P value | 0.950 | 0.594 | 0.301 |
| Overall AS |  |  |  |
| Estimate (95% CI) | 0.001 (-0.001, 0.003) | 0.002 (-0.001, 0.006) | -1.2E-04 (-3.0E-04, 5.8E-05) |
| P value | 0.398 | 0.241 | 0.188 |
| AS in female |  |  |  |
| Estimate (95% CI) | -1.57E-05 (-0.003, 0.002) | 0.001 (-0.003, 0.004) | -1.0E-04 (-2.8E-04, 8.3E-05) |
| P value | 0.990 | 0.682 | 0.283 |
| AS in male |  |  |  |
| Estimate (95% CI) | 0.003 (-0.001, 0.007) | 0.004 (-0.002, 0.010) | -1.4E-04 (-4.2E-04, 1.4E-04) |
| P value | 0.183 | 0.204 | 0.317 |
| Hip OA |  |  |  |
| Estimate (95% CI) | 0.482 (0.261, 0.702) | 0.443 (0.032, 0.854) | -0.005 (-0.026, 0.015) |
| P value | 1.86E-05 | 0.035 | 0.613 |
| Knee OA |  |  |  |
| Estimate (95% CI) | 0.085 (-0.090, 0.259) | -0.086 (-0.450, 0.279) | 0.007 (-0.011, 0.025) |
| P value | 0.341 | 0.646 | 0.419 |

CI = confidence interval; P value = p-value of the causal estimate.

Table 9. MR-PRESSO analysis for genetic associations between serum SHBG concentration and all the outcomes

| Outcome | Estimate (95% CI) | P value |
| --- | --- | --- |
| Overall OA | 0.082 (-0.032, 0.196) | 0.178 |
| OA in female | 0.005 (-0.001, 0.011) | 0.086 |
| OA in male | 0.002 (-0.002, 0.006) | 0.372 |
| Overall RA | 0.003 (-0.001, 0.007) | 0.072 |
| RA in female | 0.005 (0.001, 0.009) | 0.069 |
| RA in male | 0.001 (-0.003, 0.005) | 0.516 |
| Overall AS | 9.4E-05 (-0.002, 0.002) | 0.926 |
| AS in female | -0.001 (-0.003, 0.001) | 0.355 |
| AS in male | 0.001 (-0.003, 0.005) | 0.440 |
| Hip OA | 0.353 (0.145, 0.561) | 0.006 |
| Knee OA | 0.009 (-0.158, 0.176) | 0.917 |

For the knee OA analysis, 12 SNPs were included since rs17496332 was identified as an outlier and was removed. CI = confidence interval; P value = p-value of the causal estimate.

Table 10. IVW analysis when removing rs780093

| Outcome | Effect (95% CI) | P value |
| --- | --- | --- |
| Overall OA | 1.077 (1.000, 1.161) | 0.050 |
| OA in female | 1.006 (1.000, 1.011) | 0.032 |
| OA in male | 1.002 (0.998, 1.006) | 0.301 |
| Overall RA | 1.004 (1.000, 1.007) | 0.024 |
| RA in female | 1.006 (1.001, 1.011) | 0.025 |
| RA in male | 1.001 (0.997, 1.005) | 0.474 |
| Overall AS | 1.001 (0.999, 1.002) | 0.495 |
| AS in female | 0.999 (0.997, 1.001) | 0.462 |
| AS in male | 1.002 (0.999, 1.005) | 0.174 |
| Hip OA | 1.383 (1.181, 1.620) | 5.81E-05 |
| Knee OA | 1.011 (0.891, 1.146) | 0.870 |

Effect = odds ratio of the estimates of causal associations between SHBG levels and outcomes; CI = confidence interval; P value = p-value of the causal estimate.
